# Supplementary material for: Phylogenetic comparative analysis of the cerebello-cerebral system in 34 species highlights primate-general expansion of cerebellar crura I-II
Source: Commun Biol. 2023 Nov 22;6:1188. doi: 10.1038/s42003-023-05553-z (PMC10665558; doi:10.1038/s42003-023-05553-z)
Supplement: Supplementary file 2 — Supplementary Information [file 42003_2023_5553_MOESM2_ESM.pdf]

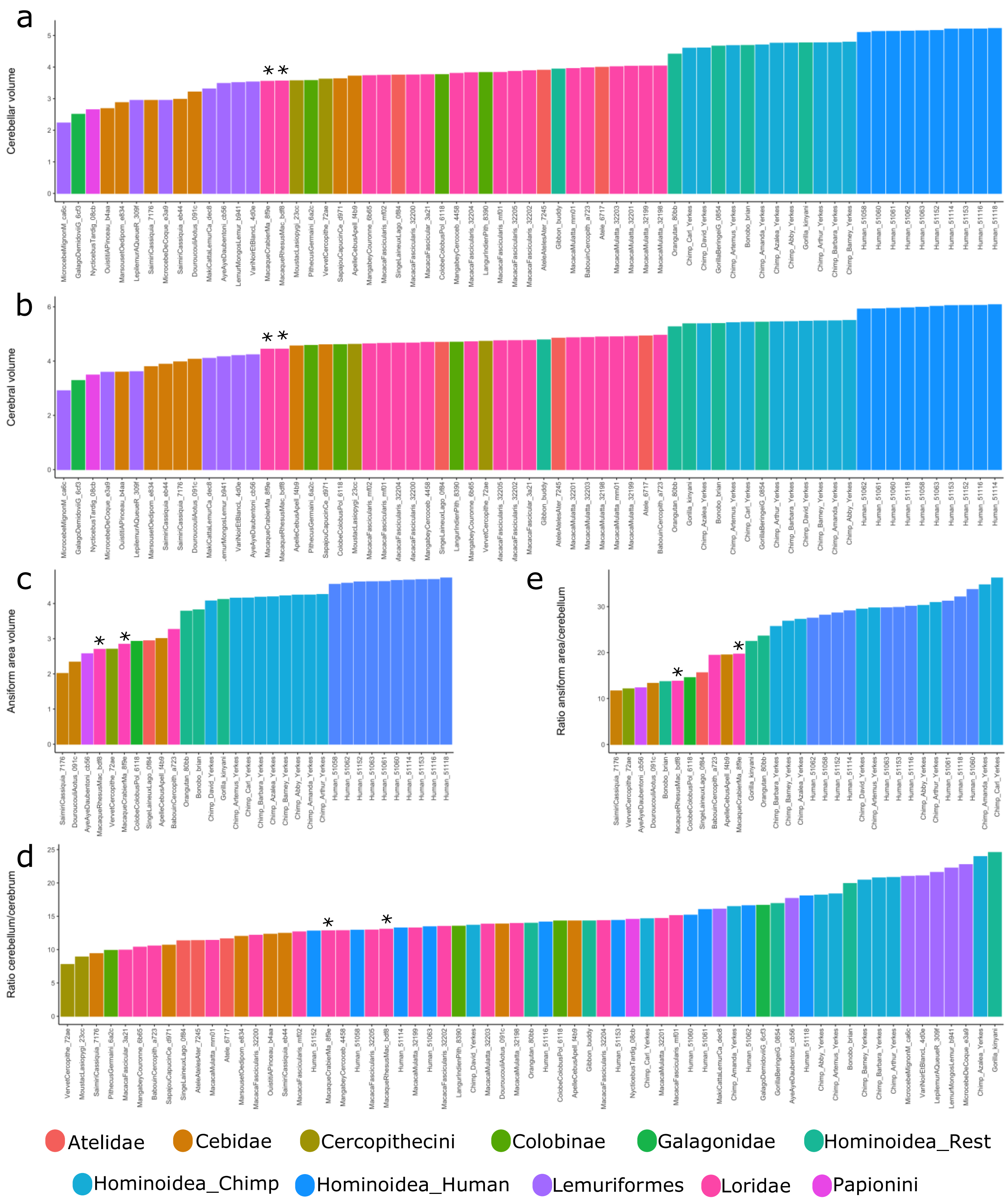

Supplementary Figure 1: Absolute and relative traits per clade. Traits are ordered based on size and colored by clade. Hominoidea are split into humans, chimpanzees, and remaining species (see legend). Absolute traits **(a-c)** (in mm^3, log_{10}-transformed) were systematically largest in apes and specifically humans. Ordinal cerebellar-to-cerebral volume ratios **(d)** (in percentages) were spread more across clades. Ansiform-to-cerebellum **(e)** volume ratios appeared especially large in great apes. Spread of both ratios are expected outcomes of isometric **(d)** and hyper-allometric **(e)** scaling. Full specimen IDs are provided on the x-axis. Two specimens that were excluded based on outlying values are marked by asterisks in all subfigures.

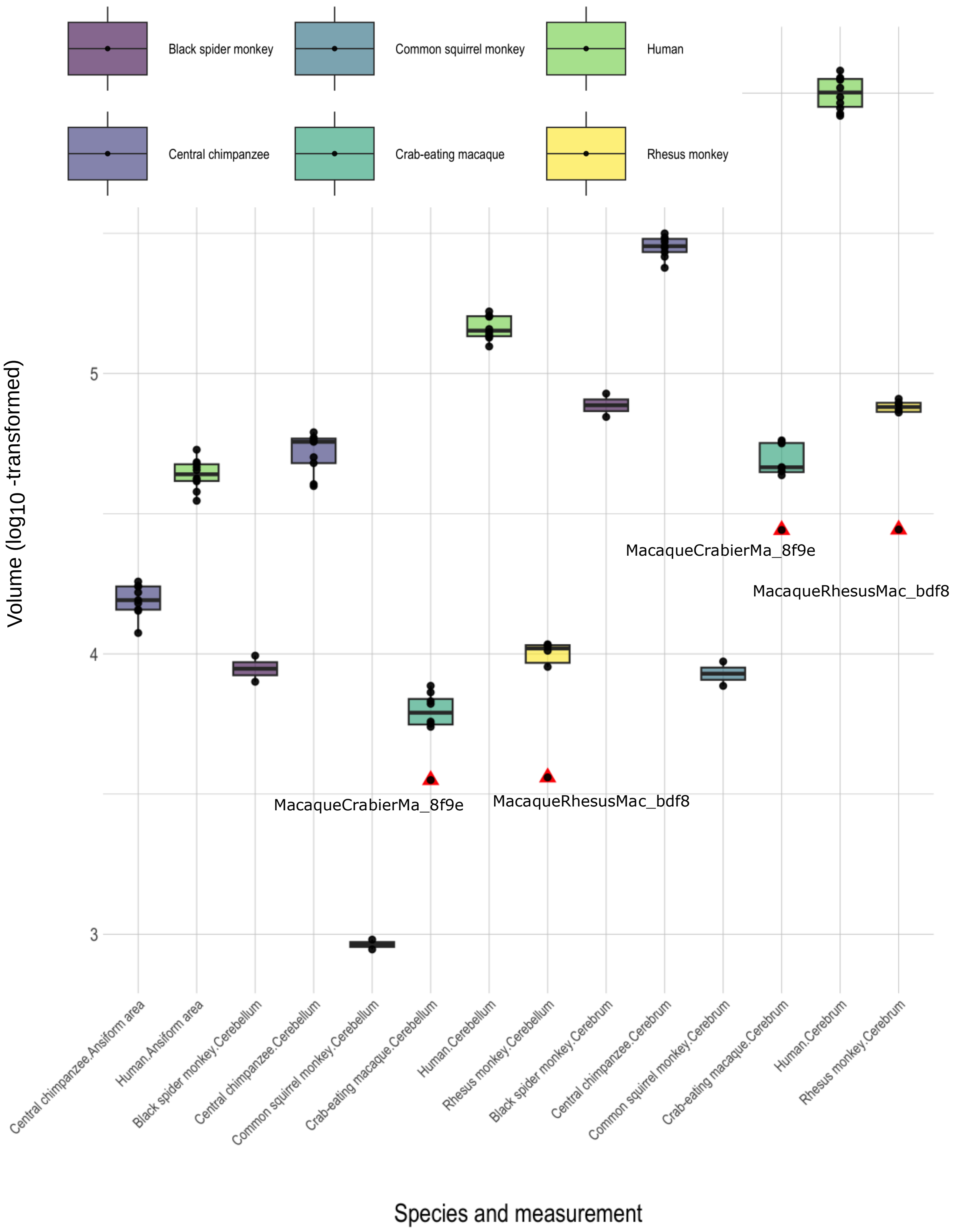

Supplementary Figure 2: Outlier detection for absolute volumes. Box plots illustrate data spread for log_{10}-transformed absolute volumes (in mm^3) for which multiple specimens (≥4) were available. For every boxplot, the colored boxes describe the interquartile range (IQR) between quartile 1 (Q1) and quartile 3 (Q3). The line within the IQR describes the median. Minimal and maximal observations are demarked by lines extending from the main box to the respective observations. Intraspecific ansiform data were available for humans and chimpanzees only. Two specimens, a crab-eating and a rhesus macaque, were identified as outliers based on exceptionally low cerebellar and cerebral volumes. Distribution shows the evolutionary scale of investigation: absolute traits show only occasional overlap between species.

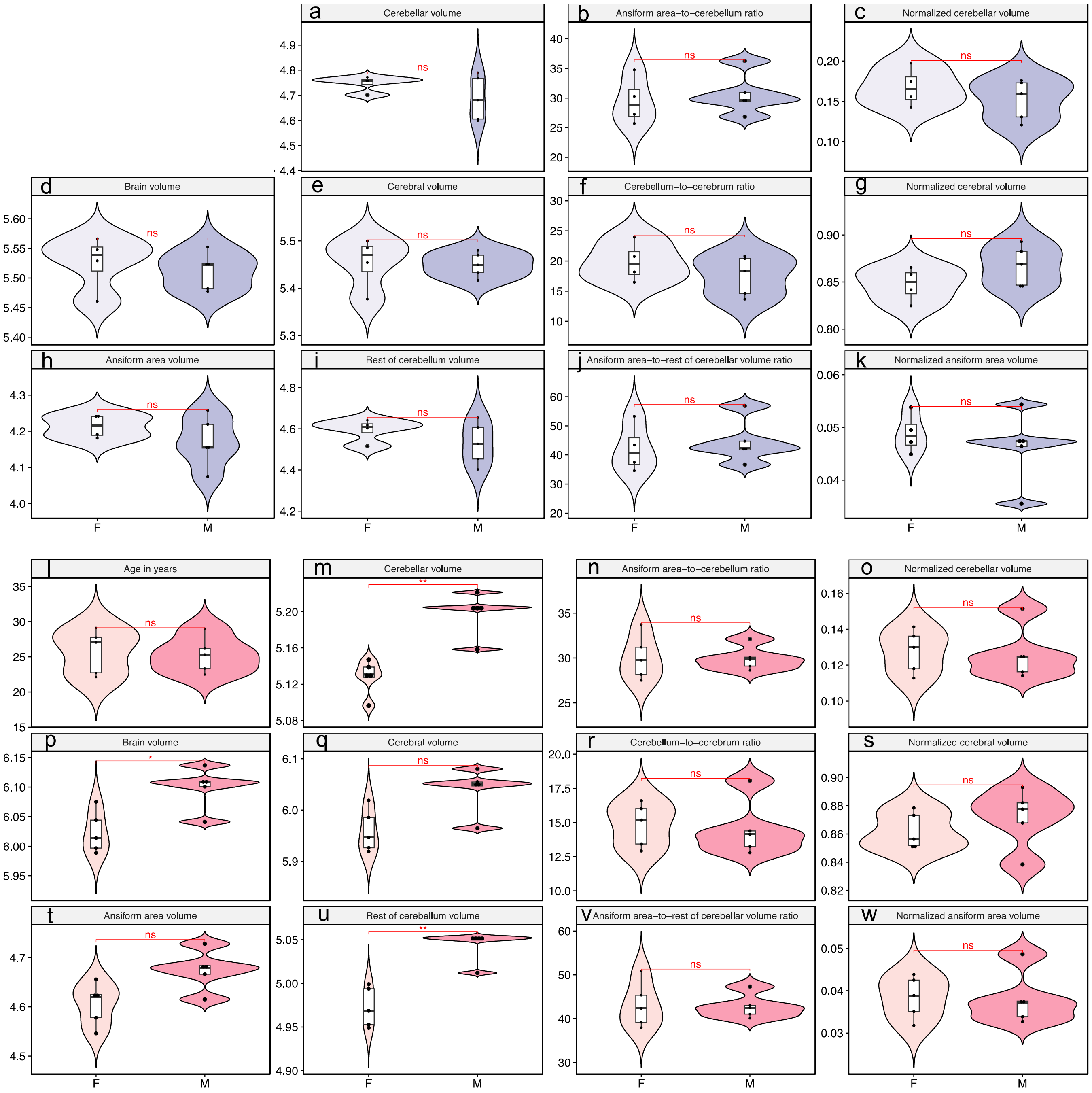

Supplementary Figure 3: Human trait distribution shows influence of sex for absolute but not relative traits. Another frequent concern about comparative data is that sex is usually not accounted for. Our analyses are limited by this uncertainty as well. To get perspective on how specimens’ sex may have mattered to the results of our study, we tested if distribution of traits differed between sexes for chimpanzees **(a-k)** and humans **(l-w)**. For every boxplot, boxes describe the interquartile range (IQR) between quartile 1 (Q1) and quartile 3 (Q3). The line within the IQR describes the median. Minimal and maximal observations are demarked by lines extending from the main box to the respective observations. Plots are overlaid with standard violin plots to aid visualization. First, we note that the chimpanzee data was likely matched data between the sexes as evidenced by highly similar brain volume **(d)** between females and males. This likely caused us to find no statistically significant difference for any other trait either. In humans, there was a significant difference in brain volume **(p)**, mirrored in differences in the other absolute traits **(m,p,q,t,u)**, some of which were significant **(m,p,u)**. However, ratios between brain components **(n,r,v)** and volumes normalized against brain volume **(o,s,w)** showed only minimal differences in distribution. Together this suggest that using specimens as

(continues)

Supplementary Figure 3: (continued) their own internal control – by regressing volumes within brains – could provide resilience to sex differences, a major driver of intraspecific variability that usually is not accounted for. Brain components may be expected to scale similarly across sexes within a species. Due to this proportionate scaling, sex of the specimens included is expected to have minimal effect on allometric scaling across the primate sample. ns = non-significant; * $p < .05$; and ** $p < .01$.

Supplementary Table 1: Ancestral character estimations (ACEs) for absolute and relative traits. ACEs are provided for cerebellar, cerebral, and ansiform volumes (in mm^3), as well as cerebellum-to-cerebrum and ansiform-to-cerebellum ratios (in percentages). Note that ratios are provided merely to show how wide-ranging volume fractions may result from primate-general allometry. ACEs incorporated intraspecies variation when multiple observations (≥4) were available **(main text, Table 1)** and were constructed from the variance-covariance matrix derived from the 34-species consensus phylogenetic tree **(main text, Figure 1)**. Estimations for absolute traits are rounded to the nearest integer, and estimations for ratios to the second decimal. Full, unrounded estimations can be found on GitHub. Internal node numbers refer to nodes in the 34-species tree **(main text, Figure 1)**.

Internal node	*Cerebellar volume*	*Cerebral volume*	*Ansiform area volume*	*Cerebellum-cerebrum ratio*	*Ansiform area-cerebellum ratio*
35	1,856	11,968	438	16.02	15.74
36	3,686	26,789	724	14.26	17.05
37	7,928	58,707	1,516	14.02	19.01
38	6,361	52,050	1,101	12.61	17.92
39	5,966	53,030	1,072	11.51	17.91
40	4,864	48,373	804	10.19	16.83
41	6,349	55,347	1,194	11.70	18.34
42	6,411	56,221	1,249	11.59	18.52
43	7,183	62,380	1,453	11.62	18.96
44	7,680	60,170	1,432	13.00	19.08
45	5,787	47,060	902	12.64	17.17
46	5,513	45,618	799	12.41	16.70
47	16,614	110,301	3,529	15.57	21.55
48	26,174	163,928	5,944	16.43	23.33
49	48,447	282,748	11,944	17.41	25.85
50	50,559	259,653	12,317	19.44	25.13
51	60,243	360,494	15,400	16.96	27.11
52	50,191	285,005	11,645	17.88	25.57
53	2,251	17,856	385	12.92	15.33
54	5,320	43,875	882	12.61	17.00
55	2,015	15,991	344	12.89	15.08
56	1,894	14,960	327	12.94	15.02
57	1,324	10,433	258	13.00	14.83
58	2,055	16,495	339	12.70	14.92
59	4,060	31,774	659	13.02	16.67
60	1,418	8,727	360	16.70	15.23
61	1,483	8,592	382	17.72	15.12
62	1,239	6,518	423	19.35	15.90
63	976	5,014	412	19.76	16.40
64	513	2,588	344	20.20	16.97
65	2,047	10,678	485	19.42	15.15
66	2,242	11,874	459	19.15	14.57
67	674	4,215	197	16.20	14.22

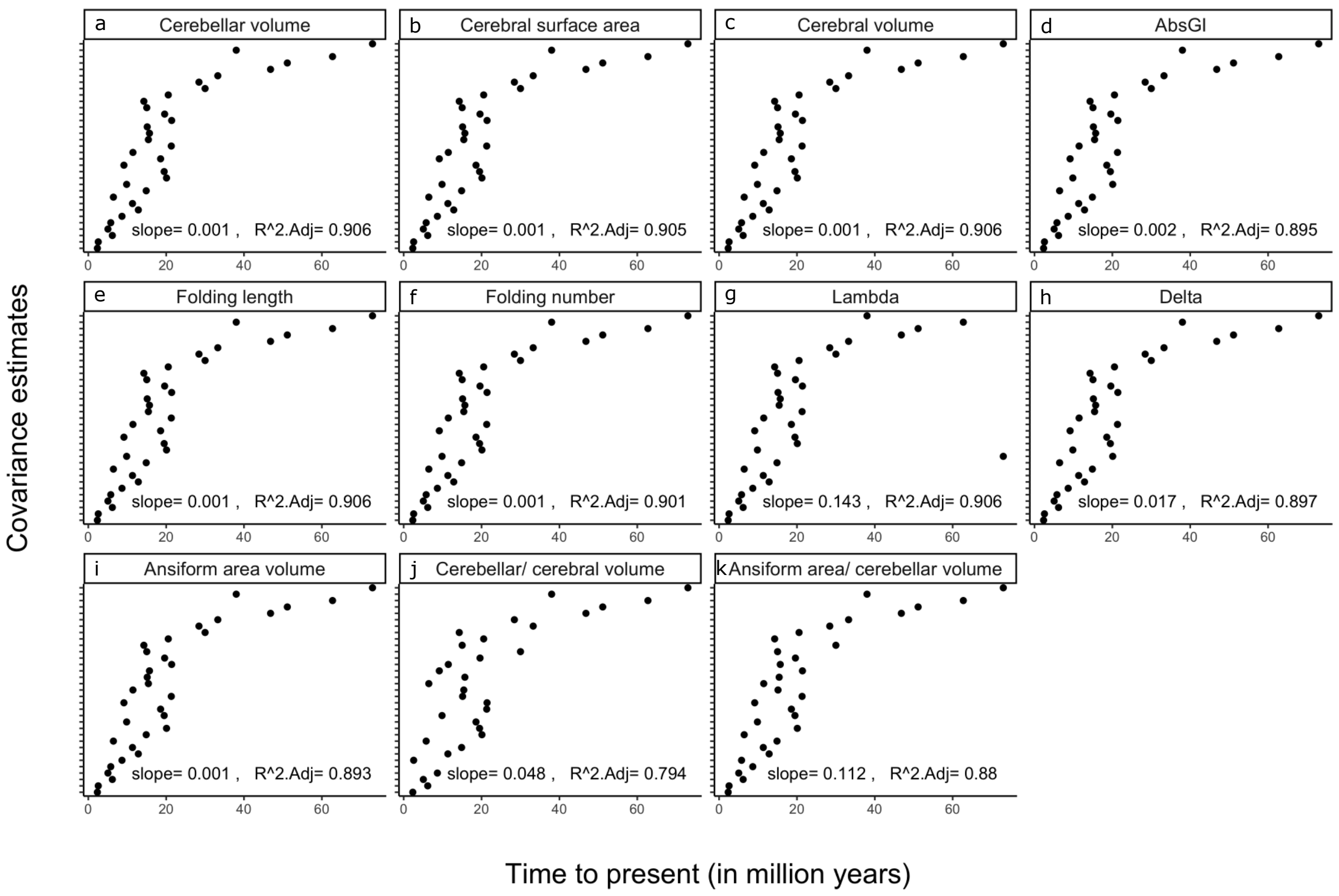

Supplementary Figure 4: Uncertainty at ancestral nodes. To explore uncertainty of ancestral character estimations (ACEs) at ancestral nodes, covariance estimates at each internal node were correlated with time to present. For all measures **(a-k)**, including those from **Heuer et al. (2019) (b-h)**, covariance correlated strongly with evolutionary time. In the measurements reported in the current study **(a,b,i-k)**, ACEs were thus more uncertain for early nodes than for more recent ones, as expected.

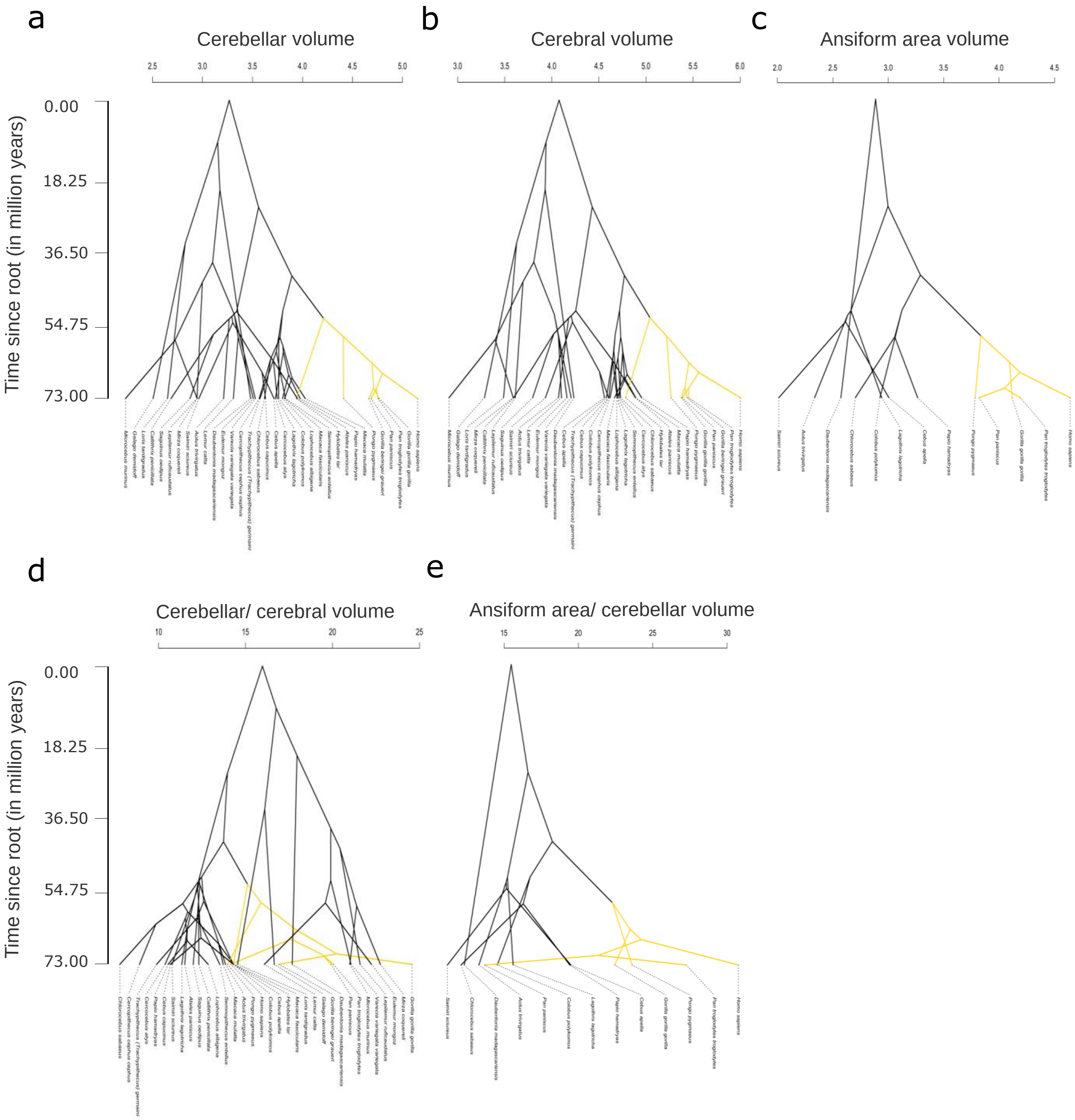

Supplementary Figure 5: Phenograms for absolute and relative traits. Alternative visualizations of traits in extant species and ancestral character estimations (ACEs), that emphasize trait values specifically. The ape-clade is colored yellow. Absolute volumes (in mm^3, log$_{10}$-transformed) **(a-c)** separated apes from non-apes, with *Hylobates lar* (common gibbon) as exception. Cerebellum-to-cerebrum ratios (in percentages) **(d)** were more mixed between apes and non-apes. Except for *Pan paniscus* (bonobo), ratios of ansiform-to-cerebellum **(e)** strongly separated apes from non-apes. Cerebellar-to-cerebral volume ratio reconstruction highlights how isometric scaling leads to a mixed distribution of ratios. Apes' general positive deviation from allometry **(main text, Figure 3a)** causes them to have ratios tending towards the higher side **(d)**. Conversely, ansiform-to-cerebellar ratios are caused by a strong hyper-allometry, that leads to large-brained primates (i.e., great apes) having much more impressive volume ratios **(e)**.

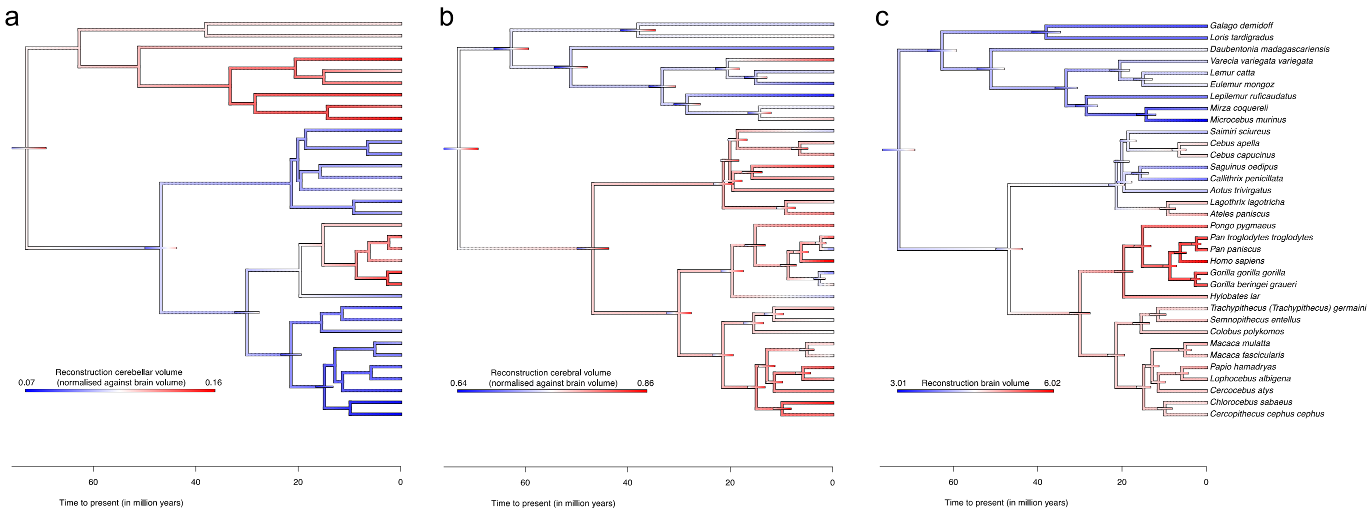

Supplementary Figure 6: Cerebella take up a larger part of the brain in lemurs and apes. To further tease apart cerebello-cerebral scaling relationships across strepsirrhines and haplorhines, we examined how traits normalized to brain volume may be distributed across our tree. These normalized volumes show how interacting allometries may lead to proportional changes between cerebellum and brain **(a)** or cerebrum and brain **(b)** between strepsirrhines and haplorhines. The ratios are not to be interpreted functionally, unlike the allometries described in the **main text, Figure 3**. **(a)** The ancestral character estimation (ACE) for normalized cerebellar volume shows a highly similar relationship as the cerebellar-cerebral ratio ACE **(main text, Figure 2c).** Ape and lemur cerebella occupy a remarkably high percentage of total brain volume. As evidenced by **(b)**, strepsirrhines (only lemurs in our data) show a characteristically smaller cerebrum relative to whole brain volume. **(c)** Lemurs are small-brained primates, but this seems irrelevant to the fraction of cerebellum-to-cerebrum **(Supplementary Figure 9b)**. Instead, cerebellar-to-cerebral scaling may reflect different cerebellar and cerebral scaling principles in strepsirrhines and haplorhines. As evidenced by **(main text, Figure 4)** and supported by **(a, b)**, a potentially heightened intercept of cerebellar scaling in strepsirrhines is a potential candidate explaining increased relative cerebellar volumes in lemurs. Normalized volumes are given in fractions **(a,b)** whereas brain volume represents $\log_{10}$-transformed mm^3 **(c)**.

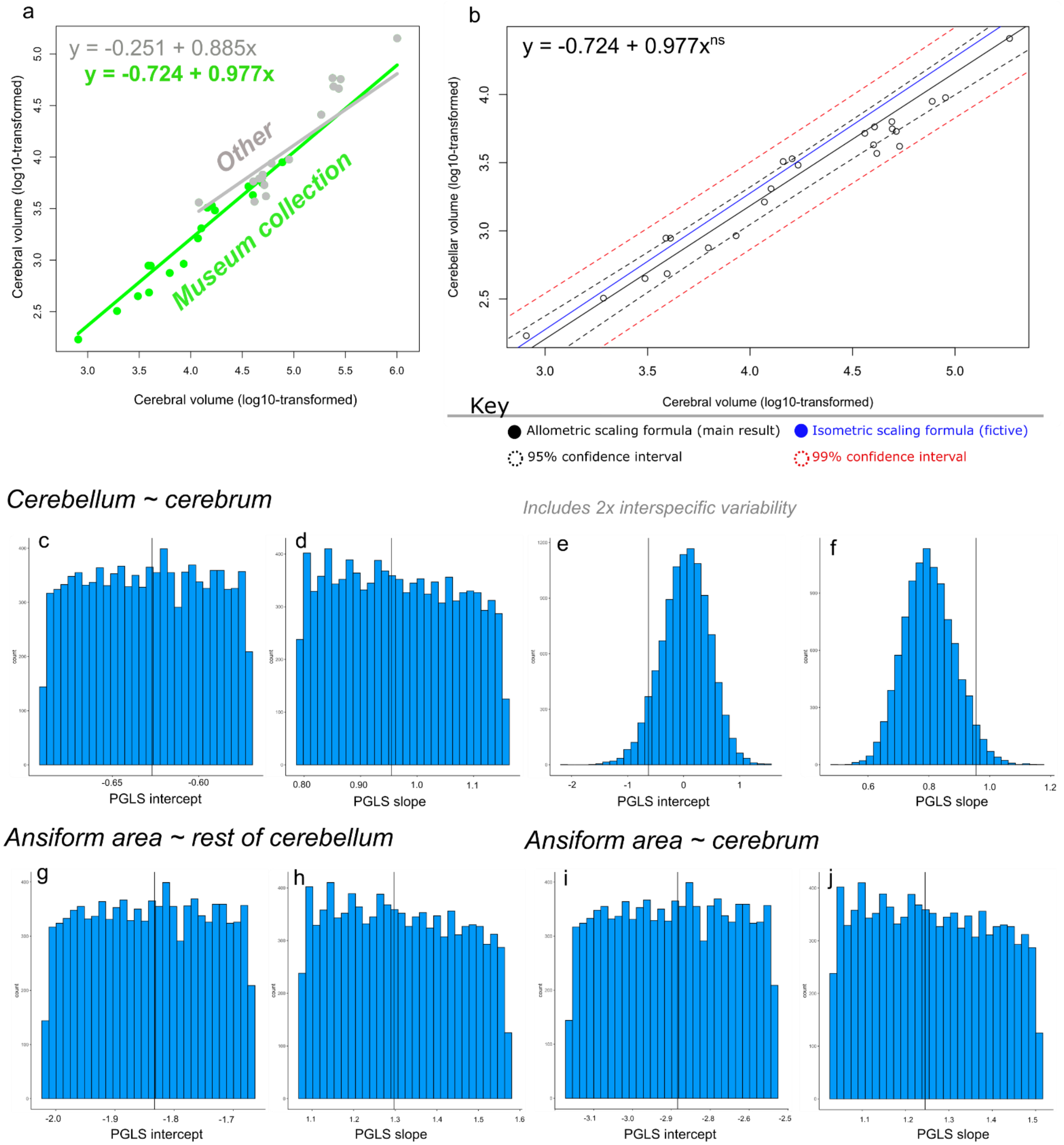

Supplementary Figure 7: PGLS results are resilient to shrinkage between brains but tissue shrinkage may matter. To obtain a large primate dataset, data was collated from diverse provenances. This may raise concerns about comparability of the data. Although our main analyses are designed to be less sensitive to variability across brains – but rather within them – it is important to consider how unaccounted for shrinkage or other sources of interspecific variability affect PGLS analyses. In **(a)** we split our data into two groups: i.) data from the National Museum of Natural History (MNHN), and ii.) other (non-MNHN) data. The MNHN is our main source of data, and includes brains conserved for several decades. They are expected to have shrunk by a similar factor, and more than the other data. Running PGLS across groups showed significant scaling differences between them, when we allowed slope and intercept to vary simultaneously. The non-MNHN slope was appreciably reduced. The MNHN PGLS **(b)** showed a non-significant hypo-allometric scaling relationship that was comparable to the main PGLS, albeit with a slightly increased slope. Since scaling in MNHN data appeared different, we explored how unaccounted for shrinkage may affect PGLS

(continues)

Supplementary Figure 7: (continued) scaling. Thus, we simulated 10.000 PGLS analyses, with simulated shrinkage difference across tissues (a factor between 0.91 and 1.1) **(c,d,g-j)** and between brains (2.0x interspecific variability) **(e,f)**. In **(c,d)** we show how the intercept and slope of PGLS analyses changed when introducing random shrinkage differences between cerebellum and cerebrum, showing that unequal shrinkage may cause differences in PGLS interpretation. In contrast, ansiform hyper-allometric scaling, both relative to the rest of the cerebellum **(g,h)** and cerebrum **(i,j)** was resilient to introducing this random shrinkage of up to 10% between tissues. To assess the potential effect of unaccounted for shrinkage across brains in combination with unaccounted for natural intraspecific variability, we introduced random transformations between factors of 0.5 and 2.0 between brains (species). Running 10.000 simulations of PGLS analysis between cerebellum and cerebrum shows that there is a large potential spread of intercept **(e)** and slope **(f)**, centering at approximately 0.0 and 0.8, respectively. Our main PGLS results fall on the high end of the 95% confidence interval, thus appearing more likely to overestimate, rather than underestimate, the slope. These results fall in line with our replication in the Stephan dataset **(main text, Figure 6)** and PGLS analysis excluding literature-based outliers **(Supplementary Figure 8)**. Together, simulations illustrated that PGLSs were robust to large interspecific differences based on shrinkage and intraspecific variability, but that exact scaling formulae may be influenced by tissue-specific shrinkage differences especially. For the cerebellum-cerebrum PGLS, this shrinkage may alter the ultimately conclusion, whereas ansiform hyperscaling appears robust to even this shrinkage. NS = non-significant.

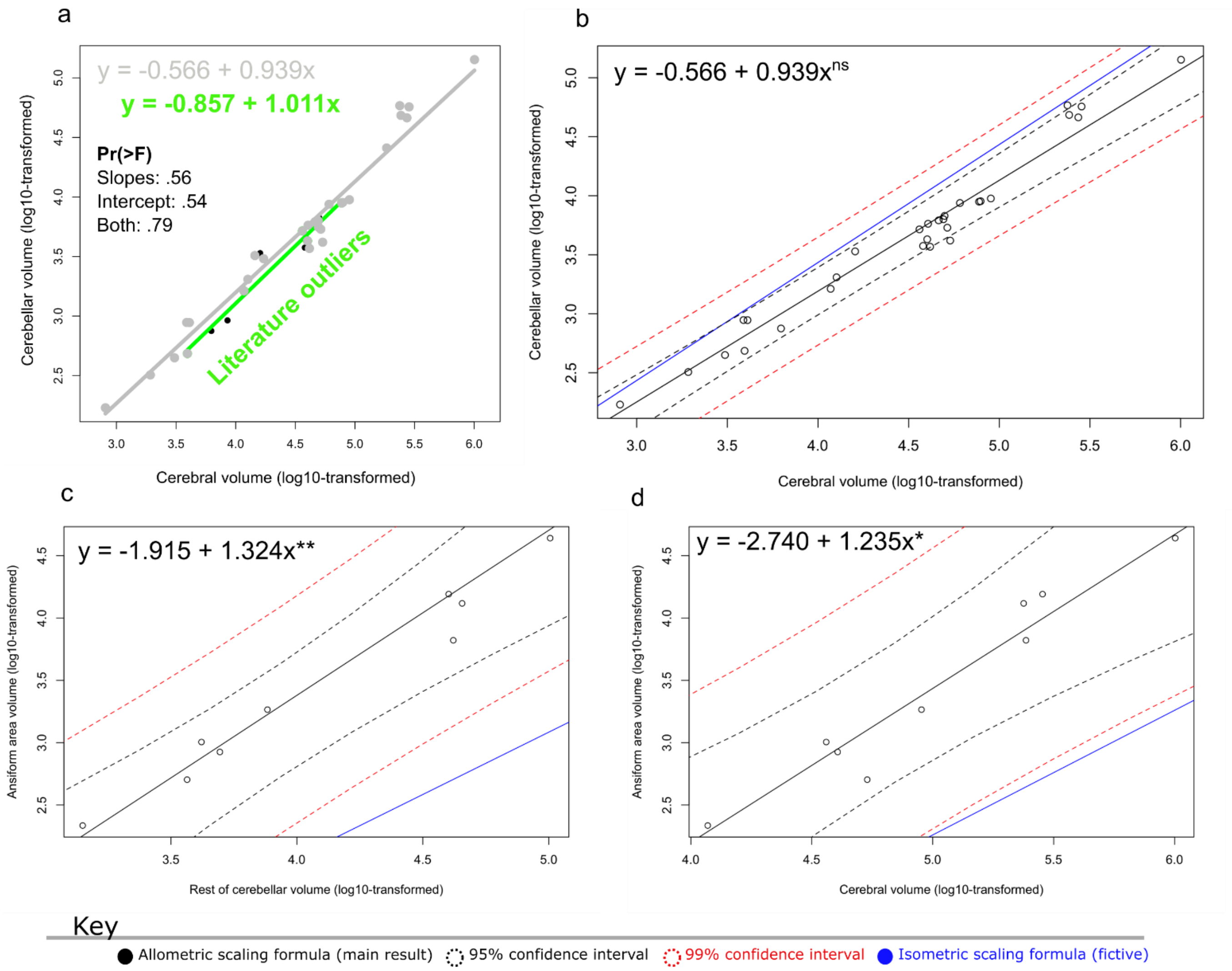

Supplementary Figure 8: Main PGLS results may be bolstered by removing literature-identified outlier values. Because (primate) comparative studies inevitable face a tradeoff between data breadth and depth, it is difficult to reliably uncover outlier values within-sample. In this conservative replication of our main PGLS analysis, we removed any species whose median cerebellar volume were more than twice as small or large as previously reported volumes. Because individual data points are usually not available in the literature, we could not exclude species based on intraspecific distributions and instead decided on this method based on maximal assumed intraspecific variability . **(a)** We first split up the data into subsets: i.) data inconsistent with the literature and ii.) data consistent with, or not previously reported in the literature (to our knowledge). Running PGLS for both groups and testing if there was a difference in intercept, slope, or both revealed that there were no significant differences. However, the scaling formulae reveal how the potential outlier values in our data may have led to a steeper slope in the main analysis. **(b)** PGLS regression, removing the outlier values, revealed a reduced slope of cerebellar-to-cerebral scaling relative to our main analysis. This scaling was more consistent with the Stephan data analysis and trended toward being a significant hypo-allometry. **(c-d)** Removing the outlier data showed a slightly steeper slope for the ansiform regressed against rest of cerebellar volume **(c)** and slightly reduced slope for the ansiform regressed against cerebral volume. These results show that our main results were robust to potentially included outlier values. If anything, our main results underestimated the hypo-allometry of the cerebellum relative to the cerebrum, and the hyper-allometry of the ansiform relative to the rest of the cerebellum. These results were consistent with our simulation analysis (**Supplementary Figure 7**). ns = non-significant.

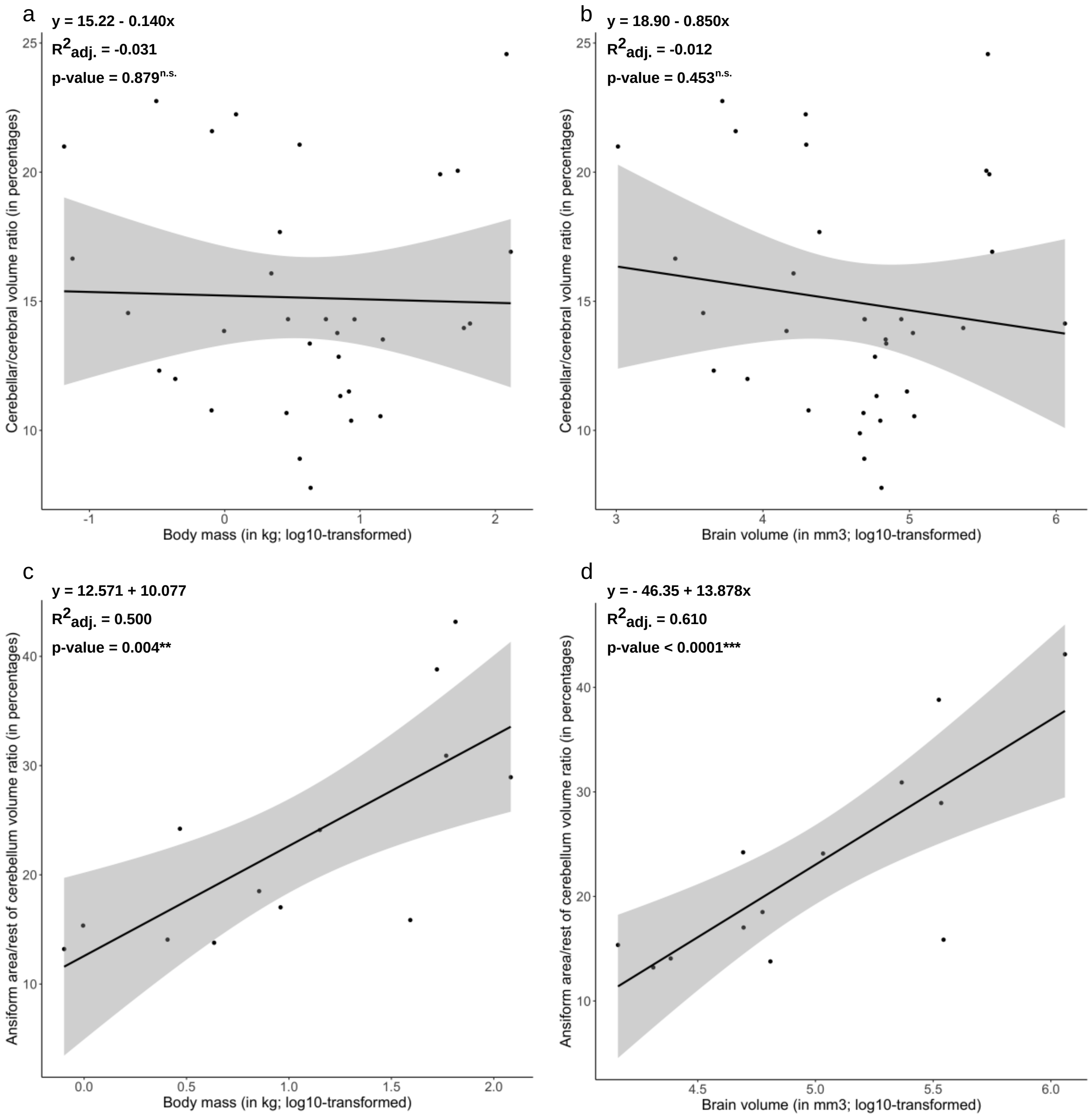

Supplementary Figure 9: Ansiform hyper-allometry leads to a direct relationship between its volume fraction and brain and body size. We explored whether cerebellum-to-cerebrum **(a, c)** and ansiform-to-rest of cerebellum **(b,d)** ratios could be directly related to body mass (in kg; $\log_{10}$-transformed) or brain volume (in mm^3; $\log_{10}$-transformed). Linear regression showed no statistically significant relationship of relative (to cerebral) cerebellar volume with body mass **(a)**, nor with brain volume. However, relative ansiform volume (to rest of cerebellar volume) **(b)** showed association with body mass ($R^2_{adj.}$ = .500, p=.004**), and more strongly with brain volume ($R^2_{adj.}$ = .610, p<.0001****). This indicates that cross-species variability of relative ansiform volume can be closely predicted by species' brain volumes, and to a slightly lesser extent by species' body mass. This association results directly from ansiform hyper-allometry. Adj. = adjusted; n.s. = non-significant. Grey shading represents 95% confidence intervals of the linear regressions.

Supplementary Table 2: R software packages used in this study. Software used for analyses or data handling in this study that are not included in base R are listed. Authors of packages or functions are accredited under source and in the main manuscript references.

**Package or *function***	Source
ape	Paradis, E. & Schliep, K. ape 5.0: an environment for modern phylogenetics and evolutionary analyses in R. *Bioinformatics* **35**, 526–528 (2019).
corrplot	Wei, T. & Simko, V. *R package 'corrplot': Visualization of a Correlation Matrix*. (2021).
data.table	https://cran.r-project.org/web/packages/data.table/index.html.
dispRity	Guillerme, T. dispRity: A modular R package for measuring disparity. Methods Ecol. Evol. 9, 1755–1763 (2018).
evomap	Smaers, JB: https://github.com/JeroenSmaers/evomap.
ggpattern	https://github.com/coolbutuseless/ggpattern.
ggplot2	Wickham, H. *ggplot2*. (Springer International Publishing, 2016). doi:10.1007/978-3-319-24277-4.
ggplotRegression	Johnston, S: https://sejohnston.com/2012/08/09/a-quick-and-easy-function-to-plot-lm-results-in-r/.
ggpmisc	Aphalo, P. *Learn R ...as you learnt your mother tongue*. (2017).
lsmeans	Lenth, R. V. Least-Squares Means: The R Package lsmeans. *J. Stat. Softw.* **69**, 1–33 (2016).
nlme	Pinheiro, J., Bates, D. & R Core Team. nlme: Linear and Nonlinear Mixed Effects Models. (2022).
nortest	https://cran.r-project.org/web/packages/nortest/index.html.
phytools	Revell, L. J. phytools: an R package for phylogenetic comparative biology (and other things). *Methods Ecol. Evol.* **3**, 217–223 (2012).
psych	Revelle, W. psych: Procedures for Psychological, Psychometric, and Personality Research. R Package Version 1.0–95. *Evanst. Ill.* (2013).
Rphylopars	Goolsby, E. W., Bruggeman, J. & Ané, C. Rphylopars: fast multivariate phylogenetic comparative methods for missing data and within-species variation. *Methods Ecol. Evol.* **8**, 22–27 (2017).
rr2	Ives, A. & Li, D. rr2: An R package to calculate R2s for regression models. *J. Open Source Softw.* **3**, 1028 (2018).<br>And:<br>Ives, A. R. R2s for Correlated Data: Phylogenetic Models, LMMs, and GLMMs. Systematic Biology 68, 234–251 (2019).
tidyverse	Wickham, H. *et al.* Welcome to the Tidyverse. *J. Open Source Softw.* **4**, 1686 (2019).

Supplementary Note 1

We did not find significant interaction with ape membership for cerebellum regressed on cerebrum: $F(Df=1, Df_{denom.}=30)=1.17$, $p=.288$. Fisher's R-to-Z transformation indicated significantly worse fit for apes (r=.75, Z=4.37, p<.0001.) and non-apes (r=.87, Z=3.00, p<.01) versus the full model (r=.97). Evidence for difference between groups became even weaker in the 13-species data: $F(Df=1, Df_{denom.}=9)=.52$, $p=.49$. The full model again better described the data (r=.94) than apes (r=.43, Z= 2.86, p<.01) or non-apes (r=.76, Z=1.70, p=.089) separately. We also found no support in the ansiform regressions on rest of cerebellar ($F(Df=1, Df_{denom.}=9)=1.87$, $p=.204$), or cerebral volume ($F(Df=1, Df_{denom.}=9)=.045$, $p=.84$). The full model outperformed partial models for cerebellar regression (full: r=.85; ape: r=.61, Z=1.19, p=.23; non-ape: r=.90, Z=.53, p=.596) and cerebral regression (full: r=.87; ape: r=.56, Z=1.57, p =.116; non-ape: r=.68, Z=1.14, p=.254). Models fit better in non-apes than in apes for rest of cerebellar ($r_{apes}=.61$, $r_{non\text{-}apes}=.90$, Z=1.72 p=.085) and cerebral regressions ($r_{apes}=.56$, $r_{non\text{-}apes}=.68$, Z=.43, p=.667), with only a trend towards significance observed for the cerebellar regression. Summarizing, all regressions were best-described by one primate-general regession.

Supplementary References

1. Paradis, E. & Schliep, K. ape 5.0: an environment for modern phylogenetics and evolutionary analyses in R. *Bioinformatics* **35**, 526–528 (2019).
2. Wei, T. & Simko, V. *R package 'corrplot': Visualization of a Correlation Matrix*. (2021).
3. Guillerme, T. dispRity: A modular R package for measuring disparity. *Methods Ecol. Evol.* **9**, 1755–1763 (2018).
4. Wickham, H. *ggplot2*. (Springer International Publishing, 2016). doi:10.1007/978-3-319-24277-4.
5. Aphalo, P. *Learn R ...as you learnt your mother tongue*. (2017).
6. Lenth, R. V. Least-Squares Means: The R Package lsmeans. *J. Stat. Softw.* **69**, 1–33 (2016).
7. Pinheiro, J., Bates, D. & R Core Team. nlme: Linear and Nonlinear Mixed Effects Models. (2022).
8. Revell, L. J. phytools: an R package for phylogenetic comparative biology (and other things). *Methods Ecol. Evol.* **3**, 217–223 (2012).
9. Revelle, W. psych: Procedures for Psychological, Psychometric, and Personality Research. R Package Version 1.0–95. *Evanst. Ill.* (2013).
10. Goolsby, E. W., Bruggeman, J. & Ané, C. Rphylopars: fast multivariate phylogenetic comparative methods for missing data and within-species variation. *Methods Ecol. Evol.* **8**, 22–27 (2017).
11. Ives, A. & Li, D. rr2: An R package to calculate R2s for regression models. *J. Open Source Softw.* **3**, 1028 (2018).
12. Ives, A. R. R2s for Correlated Data: Phylogenetic Models, LMMs, and GLMMs. *Systematic Biology* **68**, 234-251 (2019).

13. Wickham, H. *et al.* Welcome to the Tidyverse. *J. Open Source Softw.* **4**, 1686 (2019).
