# Supplementary material for: Phylogenetic comparative analysis of the cerebello-cerebral system in 34 species highlights primate-general expansion of cerebellar crura I-II
Source: Commun Biol. 2023 Nov 22;6:1188. doi: 10.1038/s42003-023-05553-z (PMC10665558; doi:10.1038/s42003-023-05553-z)
Supplement: Supplementary file 3 — Description of Additional Supplementary Files [file 42003_2023_5553_MOESM3_ESM.pdf]

Description of Additional Supplementary Files

File name: Supplementary Data 1

Description: Quality Control for Primate Brains. Data quality metrics for the magnetic resonance imaging (MRI) data used in the current study. Per-specimen MRI resolutions and signal-to-noise ratios are provided next to in vivo (relative to ex vivo) status, and in situ (relative to extracted) status.

File name: Supplementary Data 2
Description: Source data behind the graphs in the figures.
