# Supplementary material for: Phylogenetic comparative analysis of the cerebello-cerebral system in 34 species highlights primate-general expansion of cerebellar crura I-II
Source: Commun Biol. 2023 Nov 22;6:1188. doi: 10.1038/s42003-023-05553-z (PMC10665558; doi:10.1038/s42003-023-05553-z)
Supplement: Supplementary file 6 — Reporting Summary [file 42003_2023_5553_MOESM6_ESM.pdf]

nature portfolio

Corresponding author(s): Neville Magielse, MSc.
Sofie Valk, Dr.

Last updated by author(s): 01.11.2023

Reporting Summary

Nature Portfolio wishes to improve the reproducibility of the work that we publish. This form provides structure for consistency and transparency in reporting. For further information on Nature Portfolio policies, see our Editorial Policies and the Editorial Policy Checklist.

Statistics

For all statistical analyses, confirm that the following items are present in the figure legend, table legend, main text, or Methods section.

n/a	Confirmed	
☐	☒	The exact sample size (*n*) for each experimental group/condition, given as a discrete number and unit of measurement
☒	☐	A statement on whether measurements were taken from distinct samples or whether the same sample was measured repeatedly
☐	☒	The statistical test(s) used AND whether they are one- or two-sided<br>*Only common tests should be described solely by name; describe more complex techniques in the Methods section.*
☐	☒	A description of all covariates tested
☐	☒	A description of any assumptions or corrections, such as tests of normality and adjustment for multiple comparisons
☐	☒	A full description of the statistical parameters including central tendency (e.g. means) or other basic estimates (e.g. regression coefficient) AND variation (e.g. standard deviation) or associated estimates of uncertainty (e.g. confidence intervals)
☐	☒	For null hypothesis testing, the test statistic (e.g. *F*, *t*, *r*) with confidence intervals, effect sizes, degrees of freedom and *P* value noted<br>*Give P values as exact values whenever suitable.*
☒	☐	For Bayesian analysis, information on the choice of priors and Markov chain Monte Carlo settings
☒	☐	For hierarchical and complex designs, identification of the appropriate level for tests and full reporting of outcomes
☐	☒	Estimates of effect sizes (e.g. Cohen's *d*, Pearson's *r*), indicating how they were calculated

Our web collection on statistics for biologists contains articles on many of the points above.

Software and code

Policy information about availability of computer code

Data collection	We used previously acquired and reported data, please see Heuer et al., 2019 for details and find our Data Availability statement.
Data analysis	All code can be found at https://github.com/NevMagi/34primates_cerebellum. Used Open Software is mentioned in the manuscript.

For manuscripts utilizing custom algorithms or software that are central to the research but not yet described in published literature, software must be made available to editors and reviewers. We strongly encourage code deposition in a community repository (e.g. GitHub). See the Nature Portfolio guidelines for submitting code & software for further information.

Data

Policy information about availability of data

All manuscripts must include a data availability statement. This statement should provide the following information, where applicable:

- Accession codes, unique identifiers, or web links for publicly available datasets
- A description of any restrictions on data availability
- For clinical datasets or third party data, please ensure that the statement adheres to our policy

We have included this statement, including the mention that the Stephan dataset is not openly available, but was obtained through the library of our Research Institution.

Human research participants

Policy information about studies involving human research participants and Sex and Gender in Research.

Reporting on sex and gender	na
Population characteristics	na
Recruitment	na
Ethics oversight	na

Note that full information on the approval of the study protocol must also be provided in the manuscript.

Field-specific reporting

Please select the one below that is the best fit for your research. If you are not sure, read the appropriate sections before making your selection.

☐ Life sciences ☐ Behavioural & social sciences ☒ Ecological, evolutionary & environmental sciences

For a reference copy of the document with all sections, see nature.com/documents/nr-reporting-summary-flat.pdf

Ecological, evolutionary & environmental sciences study design

All studies must disclose on these points even when the disclosure is negative.

Study description	Comparative analysis of the primate cerebello-cerebral system. Goal: to assess allometric relationship between components of the system in a phylogenetic context.
Research sample	34 primates species (represented by 65 specimens, 63 after exclusion). This sample comes from an openly available sample described in Heuer et al., 2019. Primate brain data are difficult to obtain and work on a 'all-you-can-get' basis. Nonetheless, our data sample the primate tree relatively well, with sizable strepsirrhine and haplorhine samples, and representing tiny to huge primates.<br><br>We included the following data availability statement:<br><br>Data availability<br>Data availability<br>Data obtained in the current project, including volumes and ratios, but also intermediate text files and figures, are uploaded to the repository accompanying this project: github.com/NevMagi/34primates_cerebellum. Input files necessary to replicate our results can also be found here, alongside full-size figures to aid legibility. MRI data have been collated from different sources, and published Open Access89. The Stephan collection (DOI: 10.1159/000155963) was not published Open Access. Therefore, access to the data was requested by the authors through the Copy Clearance Center Marketplace and facilitated through the Central Library of Forschungszentrum Jülich GmbH. Reuse permission for these data may be requested from the publisher.
Sampling strategy	na
Data collection	na
Timing and spatial scale	na
Data exclusions	Two specimens were excluded, since they were assumed to be juvenile based on outlying cerebellar and cerebral volumes relative to their conspecifics.
Reproducibility	We reran our main analysis in a completely different sample and found generally similar findings. We additionally provide several reassuring robustness analysis in different data subsets, including simulation of unaccounted for variation. However, conclusion on isometrics versus hypo-allometric scaling between cerebellum and cerebrum remains tentative and needs follow-up, as we discuss.
Randomization	Primates were (when relevant) grouped based on the primate phylogenetic tree. Covariates such a body mass may have different effects across groupings. In strepsirrhines/haplorhines, we tease apart this effect. Generally correcting for body mass is ill-advised in comparative primatology.
Blinding	For manual segmentations, NM and MA trained their ansiform area segmentations on an out-of-sample brain, and then provided their segmentations independently.

Did the study involve field work? ☐ Yes ☒ No

Reporting for specific materials, systems and methods

We require information from authors about some types of materials, experimental systems and methods used in many studies. Here, indicate whether each material, system or method listed is relevant to your study. If you are not sure if a list item applies to your research, read the appropriate section before selecting a response.

Materials & experimental systems

n/a	Involved in the study
☒	☐ Antibodies
☒	☐ Eukaryotic cell lines
☒	☐ Palaeontology and archaeology
☒	☐ Animals and other organisms
☒	☐ Clinical data
☒	☐ Dual use research of concern

Methods

n/a	Involved in the study
☒	☐ ChIP-seq
☒	☐ Flow cytometry
☒	☐ MRI-based neuroimaging
